# Feasibility of cardiovascular magnetic resonance to detect oxygenation deficits in patients with multi-vessel coronary artery disease triggered by breathing maneuvers

**DOI:** 10.1186/s12968-018-0446-y

**Published:** 2018-05-07

**Authors:** Kady Fischer, Kyohei Yamaji, Silvia Luescher, Yasushi Ueki, Bernd Jung, Hendrik von Tengg-Kobligk, Stephan Windecker, Matthias G. Friedrich, Balthasar Eberle, Dominik P. Guensch

**Affiliations:** 1 Department of Anaesthesiology and Pain Medicine, Bern University Hospital, Inselspital, University of Bern, 3010 Bern, Switzerland; 2 Research Institute of the McGill University Health Centre, Montreal, QC Canada; 3 Institute for Diagnostic, Interventional and Paediatric Radiology, Bern University Hospital, Inselspital, University of Bern, Bern, Switzerland; 4 Department of Cardiology, Bern University Hospital, Inselspital, University of Bern, Bern, Switzerland; 5 Department of Cardiology, Heidelberg University Hospital, Heidelberg, Germany

**Keywords:** Coronary artery disease, Oxygenation-sensitive cardiovascular magnetic resonance, BOLD, Breathing maneuvers, Hypocapnia, Hypercapnia

## Abstract

**Background:**

Hyperventilation with a subsequent breath-hold has been successfully used as a non-pharmacological vasoactive stimulus to induce changes in myocardial oxygenation. The purpose of this pilot study was to assess if this maneuver is feasible in patients with multi-vessel coronary artery disease (CAD), and if it is effective at detecting coronary artery stenosis > 50% determined by quantitative coronary angiography (QCA).

**Methods:**

Twenty-six patients with coronary artery stenosis (QCA > 50% diameter stenosis) underwent a contrast-free cardiovascular magnetic resonance (CMR) exam in the time interval between their primary coronary angiography and a subsequent percutaneous coronary intervention (PCI, *n* = 24) or coronary artery bypass (CABG, *n* = 2) revascularization procedure. The CMR exam involved standard function imaging, myocardial strain analysis, T2 mapping, native T1 mapping and oxygenation-sensitive CMR (OS-CMR) imaging. During OS-CMR, participants performed a paced hyperventilation for 60s followed by a breath-hold to induce a vasoactive stimulus. Ten healthy subjects underwent the CMR protocol as the control group.

**Results:**

All CAD patients completed the breathing maneuvers with an average breath-hold duration of 48 ± 23 s following hyperventilation and without any complications or adverse effects. In comparison to healthy subjects, CAD patients had a significantly attenuated global myocardial oxygenation response to both hyperventilation (− 9.6 ± 6.8% vs. -3.1 ± 6.5%, *p* = 0.012) and apnea (11.3 ± 6.1% vs. 2.1 ± 4.4%, *p* < 0.001). The breath-hold maneuver unmasked regional oxygenation differences in territories subtended by a stenotic coronary artery in comparison to remote territory within the same patient (0.5 ± 3.8 vs. 3.8 ± 5.3%, *p* = 0.011).

**Conclusion:**

Breathing maneuvers in conjunction with OS-CMR are clinically feasible in CAD patients. Furthermore, OS-CMR demonstrates myocardial oxygenation abnormalities in regional myocardium related to CAD without the use of pharmacologic vasodilators or contrast agents. A larger trial appears warranted for a better understanding of its diagnostic utility.

**Trial registration:**

Clinical Trials Identifier: NCT02233634, registered 8 September 2014.

**Electronic supplementary material:**

The online version of this article (10.1186/s12968-018-0446-y) contains supplementary material, which is available to authorized users.

## Background

In developed countries, more than 40% of deaths are due to cardiovascular diseases, in particular coronary artery disease (CAD) [1]. As a result, a large number of diagnostic procedures are performed for identifying or ruling out significant coronary artery stenosis. Some methods include identification of anatomically or hemodynamically significant stenosis in coronary arteries, although angiography based methods involve invasive measures, while others may assess perfusion deficits, indicating reduced blood flow. Many current invasive and non-invasive diagnostic techniques use surrogate markers for ischemia, however few of these common modalities fail to directly assess tissue ischemia itself, which reflects the imbalance of oxygen supply and demand. For other techniques that can assess ischemia, such as nuclear tests, these may involve exposure to radiation. Cardiovascular magnetic resonance imaging (CMR) avoids these drawbacks, although some sequences require the use of gadolinium based contrast agents, and thus are not suitable for patients with renal failure or with known contrast media allergy [2].

Oxygenation-sensitive (OS)-CMR has been introduced to directly assess myocardial oxygenation [3, 4]. This approach does not rely on pharmacological contrast agents, radiation, or invasive measures, but is based on the signal attenuating effects of the deoxyhemoglobin (dHb) fraction in the post-capillary venules, first described in the brain [5]. Hemoglobin (Hb) has paramagnetic properties when deoxygenated, which influences the phase evolution of water protons in the tissue leading to a loss in magnetic field homogeneity. This accelerates the relaxation of the spins, causing a CMR signal decrease. Oxygenated Hb has diamagnetic properties with a small stabilizing effect on the surrounding water proton relaxation times [5]. A variety of OS sequences exist, which primarily exploit this dephasing of the CMR signal relying on T2* effects. In this study we use a modified balanced steady state free procession (bSSFP) sequence that also incorporates T2/T1 effects. In healthy vasculature, vasoactive stimuli increase blood supply without an accompanying increase in oxygen demand (luxury perfusion) reducing the local dHb fraction, and thus an increase in myocardial oxygenation can be observed [6, 7]. In the presence of coronary stenosis or microvascular dysfunction, the vasodilatory response however is not as effective, or downstream vasculature is already in a compensatory state of maximum dilation. Consequently, myocardial oxygenation will not increase to the same extent as in myocardium perfused by a non-stenotic vessel. In very severe disease, a decrease may be recorded due to an inter-coronary steal, increased oxygen demand without a compensatory increase in blood flow, or post-stenotic capillary recruitment, i.e., when vessels down-stream of a stenosis dilate with deoxygenated blood [8].

In combination with pharmacological vasodilators, OS-CMR has been successfully used to detect myocardial oxygenation abnormalities in CAD [9–11]. Recently, we have investigated the use of breathing maneuvers as an endogenous non-pharmacologic, less expensive and potentially safer vasomotor stimulus. Hyperventilation induces myocardial and cerebral vasoconstriction [12, 13] in healthy subjects, and breath-holds can have a significantly stronger vasodilatory impact on myocardial oxygenation than the gold-standard of adenosine [14]. In an animal model, breathing maneuvers combined with OS-CMR identified myocardial oxygenation deficits in the presence of an acute coronary artery stenosis [15]. The combined breathing maneuver has several advantages for a clinical application. The associated hypocapnia not only allows for a longer breath-hold immediately thereafter [16], but also for monitoring a greater range of vasoreactivity, going from vasoconstriction to vasodilation during apnea. So far, the feasibility of such breathing maneuvers during OS-CMR have not been tested in the clinical setting in patients with CAD, nor has it been assessed in these patients if the technique can unmask myocardial oxygenation abnormalities subtended to a coronary artery stenosis. In combination with other CMR techniques of cine imaging for functional measurements, and T1 and T2 mapping for tissue characterisation, this study investigates the use of a fully non-contrast exam for the use of characterising CAD.

## Methods

### Participants

The study was comprised of data from participants enrolled in a single-centre study at the University Hospital Bern, Switzerland (CADOS, Clinical Trials Identifier: NCT02233634). Twenty-six patients who had undergone a primary angiography visit were included in this analysis along with 10 young and healthy control subjects. All subjects provided informed consent prior to study enrolment.

Patients were eligible to participate in the CADOS study if there was at least one untreated vascular territory with a > 50% diameter stenosis (%DS) in quantitative coronary angiography (QCA) after primary diagnostic angiography or primary percutaneous coronary intervention (PCI), and if the CMR scan could be performed prior to the secondary revascularization procedure of a secondary PCI or a scheduled coronary artery bypass graft (CABG) surgery. A further inclusion criterion was that there was an unaffected territory of least two segments according to the American Heart Association 17-segment model with presumed normal perfusion according to the angiography, to serve as intra-subject control tissue. All participants were required to be older than 18 years, capable of providing written consent, to not be pregnant or have any CMR contraindications (including but not limited to CMR non-compatible metallic objects such as pacemakers and defibrillator leads). Patients with acute myocardial infarction within 4 weeks prior to the CMR exam, with pre-existent coronary bypass grafts, or with severe pulmonary disease were also excluded.

Healthy participants were recruited by public notification, and were required to be under the age of 35 years, non-smokers for the last six months, free of any medication that would affect the cardiac or circulatory system, and with a medical history free of cardiac or pulmonary disorders, or disorders known to affect the microvasculature.

### Quantitative coronary angiography (QCA)

QCA was performed from the angiography images obtained in the primary angiography/PCI clinical visit. The %DS of all coronary arteries ≥1.5 mm was determined by a trained cardiologist independent from the CMR analysis (QangioXA version 7.3, Medis Medical Imaging Systems, Leiden, The Netherlands). To allow a comparison between the angiography and CMR results, this reader then coded each American Heart Association segment as either 1: remote, perfused by a healthy coronary artery; 2: affected by a significant stenosis (%DS > 50%); 3: reperfused with a stent; or 4: undetermined or possibly mixed perfusion territory.

### CMR protocol

**Fig. 1:**
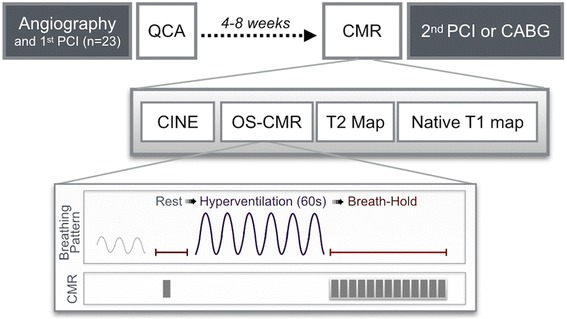
Study Protocol. Patients were recruited between staged coronary interventions or the initial diagnostic angiography and surgical revascularization therapy. After primary angiography, QCA of all vessels was performed and patients were recruited if there was at least one untreated vessel with a significant lesion (diameter stenosis; DS > 50%). For oxygen sensitive cardiovascular magnetic resonance (OS-CMR), a rest image was obtained during a short breath-hold. Participants then hyperventilated for 60s (30 breaths per minute, paced by a metronome), controlled through qualitative capnography with visual confirmation of sufficient respiratory excursions. This was immediately followed by a long breath-hold, which was imaged throughout its entire duration with repetitive OS measurements (grey boxes). Quality control of apnea also included monitoring for absence of exhaled carbon dioxide with capnography and breathing motion artifacts in the images

Prior to the CMR exam (Fig. 1), participants were asked to refrain from consuming caffeine or taking calcium antagonists or nitrates within 12 h prior to the exam. CMR imaging was performed using a clinical 3 Tesla CMR system (MAGNETOM Skyra™, Siemens Healthineers, Erlangen, Germany). The CMR included cine acquisition for ventricular function analysis, T2 and native T1 mapping, and OS-CMR during breathing maneuvers [14] in a basal and a mid-ventricular short-axis slice. The protocol involved a baseline OS-CMR cine acquisition, followed by 60s of voluntary hyperventilation at a rate of 30 breaths/min paced by a metronome and monitored with qualitative capnography through a nasal cannula line for consistency. Immediately after hyperventilation, the participant performed a long breath-hold at a comfortable exhalation level, and OS-CMR images were continuously acquired until the participant indicated the need to breathe. Blood pressure, heart rate and peripheral pulse oximetry were monitored throughout the exam. The study nurse recorded any adverse effects after the breathing maneuvers.

### Details on CMR imaging

All images were obtained at an end-expiratory breath-hold. Cine images for ventricular function analysis were obtained covering the ventricles with 7 to 10 short-axis slices using an electrocardiogram gated bSSFP sequence (TR/TE 3.3 ms/1.43 ms, temporal resolution 44.8 ms, flip angle 65°, voxel size 1.6 × 1.6 × 6.0 mm, matrix 192 × 120, bandwidth 962 Hz/Px). T1 maps, T2 maps, and OS-CMR images were obtained in 2 short-axis slices, basal and mid-ventricular. Coloured pixel-wise parametric T2 and native T1 maps were obtained for tissue characterisation. The 5(3)3-modified Look-Locker sequence was used for T1 mapping, (TR/TE 281 ms/1.12 ms, flip angle 35°, voxel size 1.4 × 1.4 × 8.0 mm, bandwidth 1085 Hz/Px). T2 maps were generated after acquiring three single-shot gradient echo images (TR/TE 207 ms/1.32 ms, flip angle = 12°; voxel size 1.9 × 1.9 × 8.0 mm, bandwidth 1184 Hz/Px with T2 preparation times of 0 ms, 30 ms, and 55 ms). OS-CMR was imaged using a triggered balanced steady-state free precession sequence (TR/TE 3.4 ms/1.70 ms, flip angle 35°, voxel size 2.0 × 2.0 × 10.0 mm, matrix 192 × 120, bandwidth 1302 Hz/Px) [14].

### CMR image blinding & analysis

**Fig. 2:**
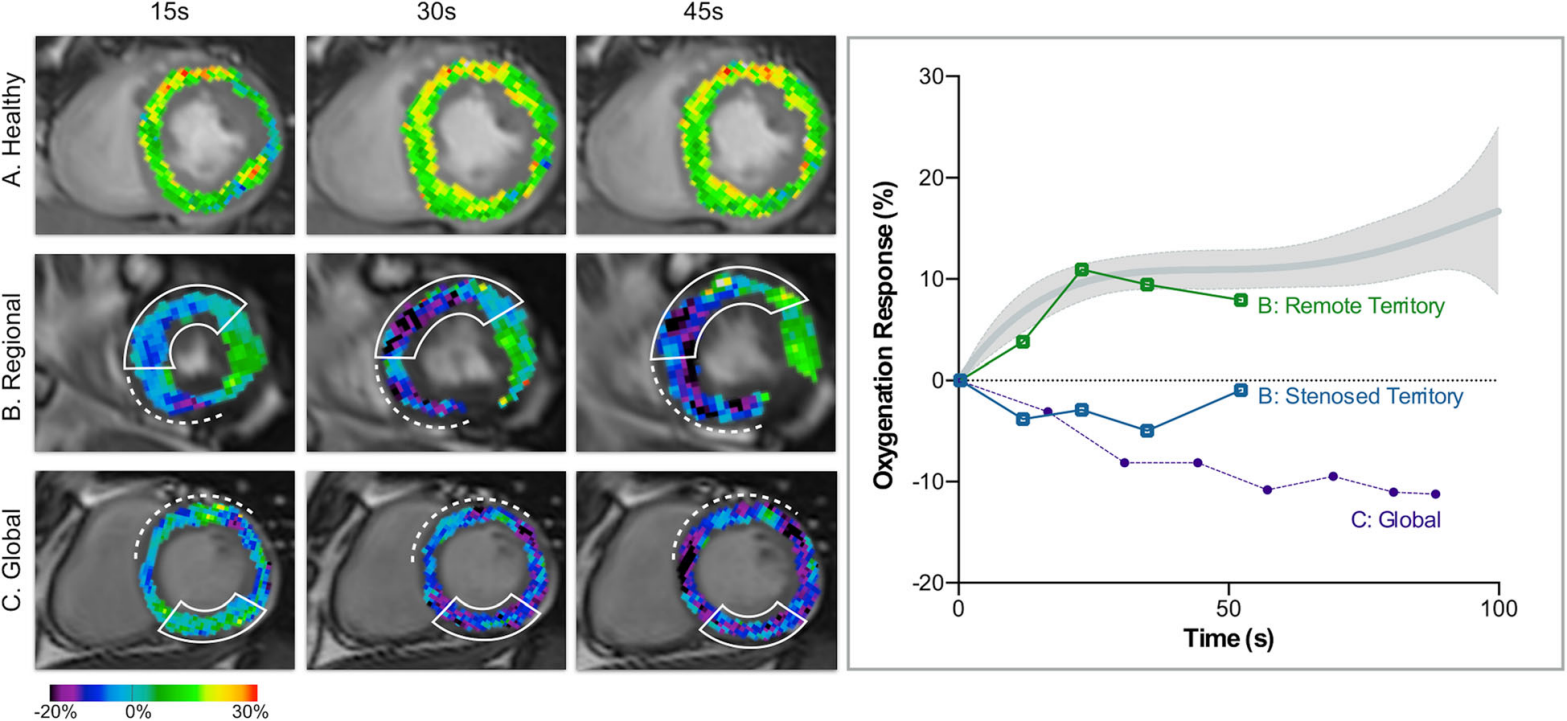
Myocardial oxygenation response throughout the breath-hold. *Left panel, top row* (**a**): In healthy subjects, myocardial oxygenation increases rapidly at the beginning of the breath-hold and is maintained throughout apnea, as shown in a healthy male as well as by the mean response of all healthy subjects (right panel, grey shaded graph). *Left panel, middle row* (**b**): OS images show a regional response (square) in a coronary artery disease (CAD) patient, whose myocardial oxygenation decreased in territory subtended to left anterior descending (LAD) stenosis (marked with a solid lined box) and in territory reperfused via a stented right coronary artery (RCA) (marked with dotted line), while the remote territory responded with an oxygenation increase (left circumflex (LCx); also note artifact rejection in a small infero-lateral region). *Left panel, bottom row* (**c**): This sequence represents a CAD patient (stented LAD, RCA stenosis) with global dysfunction in whom all territories revealed an oxygenation deficit (purple dots).

The CMR images were re-coded to blind the CMR reader to the identity of the participant, group allocation and angiography results. All CMR images were analyzed quantitatively after manual tracing of epicardial and endocardial contours using cvi^42^ (Circle CVI, Calgary, Canada) by an experienced reader. Standard function parameters were calculated including left-ventricular (LV) ejection fraction (EF), cardiac index (CI), and body surface area indexed end-diastolic (EDV_I_), end-systolic (ESV_I_) volumes and myocardial mass. Using feature-tracking software, circumferential strain was calculated for the left ventricle from the cine images, excluding slices that included the outflow tract and mitral valve planes. For strain analysis, OS-CMR, T1 and T2 mapping, data were reported as a global value, and also as segmental values, based on the automatic segmentation according to American Heart Association definitions. After un-blinding, these segmental CMR results were grouped based on the QCA categorization. Relative OS signal intensity (SI) changes were reported as %-change from baseline for the hyperventilation analysis. For the breath-hold analysis, %-change was calculated for each measurement in relation to the first image acquired after hyperventilation (Fig. 2). For statistical analysis of the apneic period, the measurement with a breath-hold duration closest to 30s was used. Maps showing the oxygenation response were created post-analysis for representation purposes only, using neurolens (neurolens.org), and were masked onto the original OS cine.

### Statistical analysis

Continuous data is reported as mean ± standard deviation (SD), while categorical data is reported as frequency and percentage. Ventricular function measurements and global myocardial values were compared between the two groups using independent Student’s t-test. Correlation was assessed using Pearson’s correlation coefficient. Non-parametric equivalents were used if appropriate. Regional analysis was performed using a repeated measures univariate linear model. Additional post-hoc analysis compared myocardium subtended to a stenosed or reperfused vessel to remote myocardium, using Bonferroni correction for multiple comparisons. For assessing inter-observer reliability, the OS response of both hyperventilation and the breath-hold of ten random participants were read by a second reader and an intra-class correlation coefficient (ICC) was determined based on single measures using a two-way mixed model assessing absolute agreement for global oxygenation changes. Furthermore, intra-observer reliability was available for eight participants that were recoded and analyzed with at least six months between readings, assessed with a one-way random ICC test. Tests were performed with GraphPad Prism (version 6.0, GraphPad Software, La Jolla California USA) and SPSS (version 24, International Business Machines, Armonk, New York, USA). Results were considered statistically significant at a two-tailed *p* < 0.05.

## Results

### Participant characteristics

Healthy participants (40% females) had a mean age of 27 ± 4 years and a body mass index of 23.9 ± 2.3 kg/m^2^. CAD patients were significantly older, with chronic medication, with the indication for PCI referral shown in Table 1. All patients had at least one vessel with a significant stenosis, defined as a %DS > 50% (mean 63 ± 15%, full chronic occlusion *n* = 3). Twenty-three patients (88%) had a PCI procedure performed on one vessel during the initial visit; three patients had diagnostic angiography only and were subsequently scheduled for subsequent PCI (*n* = 1) or a CABG surgery (*n* = 2).

**Table 1:** Patient characteristics

Age, years	64 ± 11
Female	3 (12%)
Body mass index, kg/m^2^	27.2 ± 5.0
Body weight, kg	83.3 ± 16.1
Coronary Artery Disease Risk Factors	
Dyslipidemia	15 (58%)
Hypertension	14 (54%)
Diabetes Mellitus	7 (27%)
Smokers (past 6 months)	7 (27%)
Sleep Apnea Syndrome	3 (12%)
Medication	
Statins	25 (96%)
Aspirin	25 (96%)
Dual Anti-Platelet	22 (85%)
Beta-Blockers	20 (77%)
Angiotensin-converting enzyme inhibitors	12 (46%)
Angiotensin receptor blocker	6 (23%)
Calcium Channel Blocker	3 (12%)
Cath Lab Data	
Current Stenosis	26 (100%)
QCA (%DS)	63 ± 15
PCI at index angiography	23 (88%)
Reasons for PCI	
NSTEMI	13 (50%)
STEMI	6 (23%)
Unstable angina pectoris	0 (0%)
Stable angina pectoris	7 (27%)

Baseline characteristics of patient population at time of CMR exam. *QCA* quantitative coronary angiography, *DS* diameter of stenosis, *NSTEMI* Non-ST-elevation myocardial infarction, *STEMI* ST-elevation myocardial infarction, *PCI* percutaneous coronary intervention, *QCA* quantitative coronary analysis

### Global systolic function, strain and mapping of T1 and T2

**Fig. 3:**
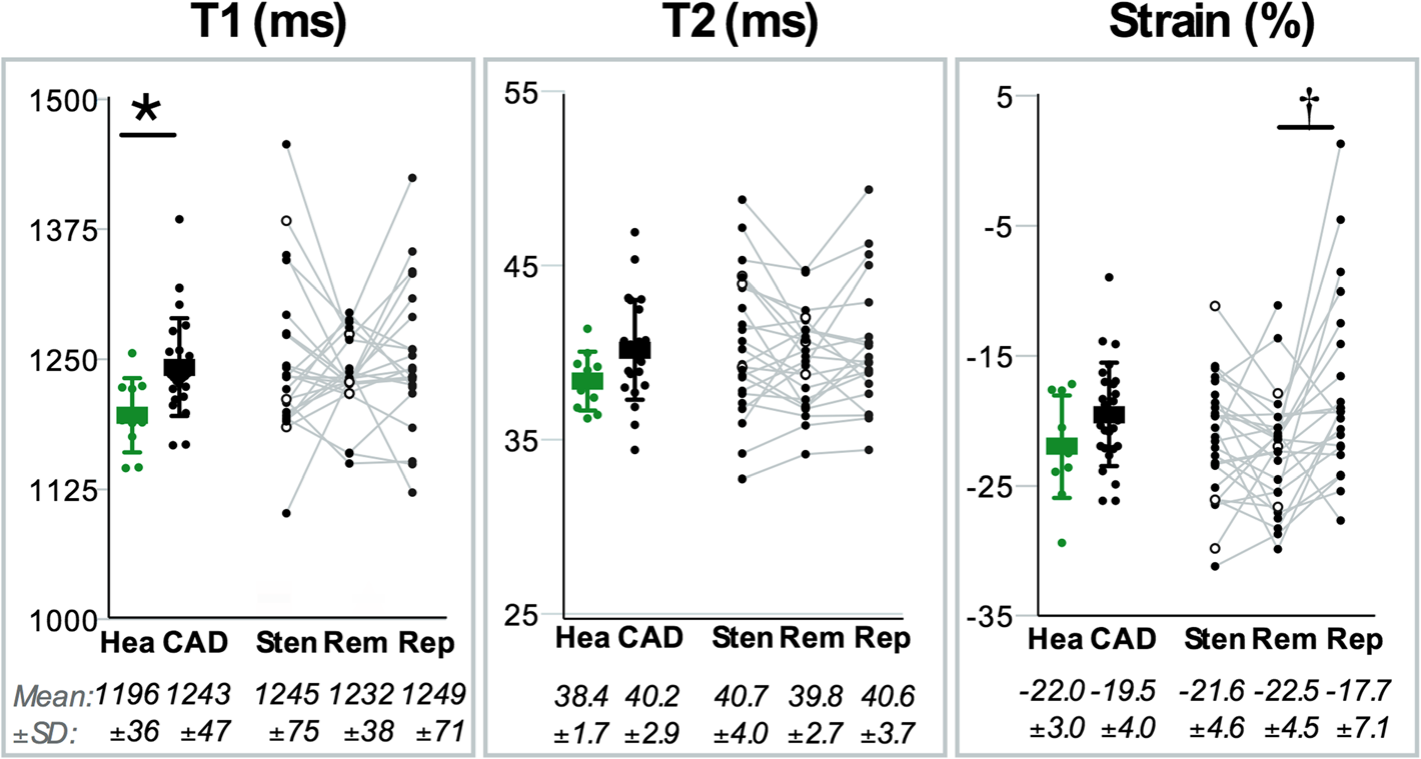
Tissue characterization and ventricular strain. Mean ± SD are shown for T1 mapping, T2 mapping and peak circumferential strain for the global responses of healthy controls (Hea) and the patient group (CAD), along with the individual regional responses of the CAD patients for territories currently affected by a stenosis (Sten) > 50%DS, remote (Rem) and recently reperfused (Rep) territories. Three patients did not have a primary PCI procedure prior to the CMR exam (white markers). *p < 0.05 between groups for global analysis. †*p* < 0.05 for regional analysis of Sten or Rep vs. Rem

Compared to healthy subjects, CAD patients had lower LVEF, stroke volume and cardiac index (Table 2). Global peak circumferential strain did not differ between groups; regional analysis revealed abnormal strain in reperfused territory, which differed significantly from remote territory, while post-stenotic territories did not (Fig. 3). Globally, CAD patients had significantly higher T1 than controls, but similar T2. Within the CAD group, there were also no significant differences in the values between the post-stenotic and reperfused territories in comparison to remote myocardium. However, some patients showed increased T1 and T2 in the segments subtended by stenotic vessels and in reperfused territories resulting in a large variation (Fig. 3).

**Table 2:** Ventricular function

	Healthy Controls	CAD	*P* Value
EDV_I_ (ml/m^2^)	85 ± 16	75 ± 15	0.090
ESV_I_ (ml/m^2^)	26 ± 5	31 ± 12	0.267
SV_I_ (ml/m^2^)	58 ± 15	44 ± 10	0.002*
EF (%)	68 ± 7	59 ± 12	0.030*
CI (L/min/m^2^)	4.1 ± 1.1	2.9 ± 0.8	0.001*
Mass_I_ (g/m^2^)	63 ± 15	68 ± 11	0.324

Mean ± SD ventricular measurements. As compared with healthy control subjects, patients with coronary artery disease (CAD) had a significantly lower stroke volume index (SV_I_), left ventricular ejection fraction (EF) and cardiac index (CI) than controls with similar left ventricular end-diastolic (EDV_I_) and end-systolic (ESV_I_) volume indexes and myocardial mass index (mass_I_). *p < 0.05 between groups

### Feasibility of the breathing maneuver

All participants successfully completed the hyperventilation component of the breathing maneuver and performed breath-holds adequately. The mean duration of the breath-hold was 75 ± 23 s in the control group and 48 ± 23 s in the patient group; two patients resumed breathing within 20s. Reported adverse effects during the breathing maneuvers were: tingling in the extremities (control: *n* = 3, CAD: *n* = 4), temporary headache (control: *n* = 2, CAD: *n* = 0), dizziness (control: *n* = 1, CAD: *n* = 0), and dry mouth (control: *n* = 0, CAD: *n* = 3). All side effects dissipated within 60s of recommencing normal breathing. The majority reported no adverse effects at all (control: *n* = 6, CAD: *n* = 17).

### Oxygenation response (OS-CMR)

OS-CMR analysis excluded 7.1% of myocardial segments, primarily because of image artifacts and thinned myocardial wall, especially in the presence of infarcts. No entire data sets were excluded, while both inter-observer (ICC: 0.96, CI: 0.91–0.99) and intra-observer (ICC: 0.99, CI: 0.98–0.99) analysis demonstrated excellent reliability.

**Fig. 4:**
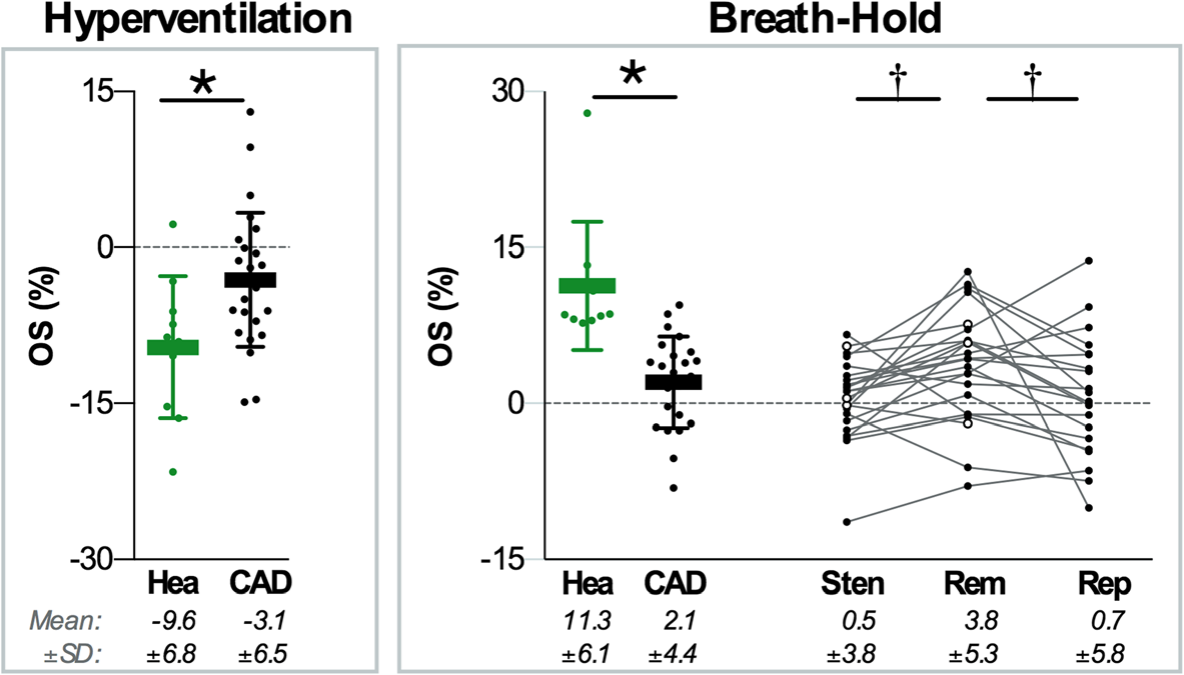
Global and regional OS-CMR. Mean ± SD are shown for the global responses of healthy control subjects (Hea) and the CAD patient group (CAD). Individual data points demonstrate that during the breath-hold (BH), the majority of patients had a lower response in the post-stenotic (Sten) and reperfused (Rep) territories than remote (Rem). Three patients did not have a primary PCI procedure prior to the CMR exam (white markers). *p < 0.05 between groups for global analysis. †*p* < 0.05 for regional analysis of Sten or Rep vs. Rem

*Global oxygenation effects:* Healthy subjects responded to hyperventilation with a global SI reduction (− 9.6 ± 6.8%), while CAD patients showed a lesser reduction (− 3.1 ± 6.5%, *p* = 0.012, Fig. 4). During the breath-hold, global myocardial oxygenation increased in the healthy subjects (+ 11.3 ± 6.1%), which was also attenuated in the CAD patients (+ 2.1 ± 4.4%, *p* < 0.001).

*Regional oxygenation heterogeneity in CAD:* There was no significant difference of the response to hyperventilation between remote (− 3.0 ± 7.1%), post-stenotic (− 2.7 ± 7.9%) or reperfused (− 2.5 ± 6.3%) segments.

**Fig. 5:**
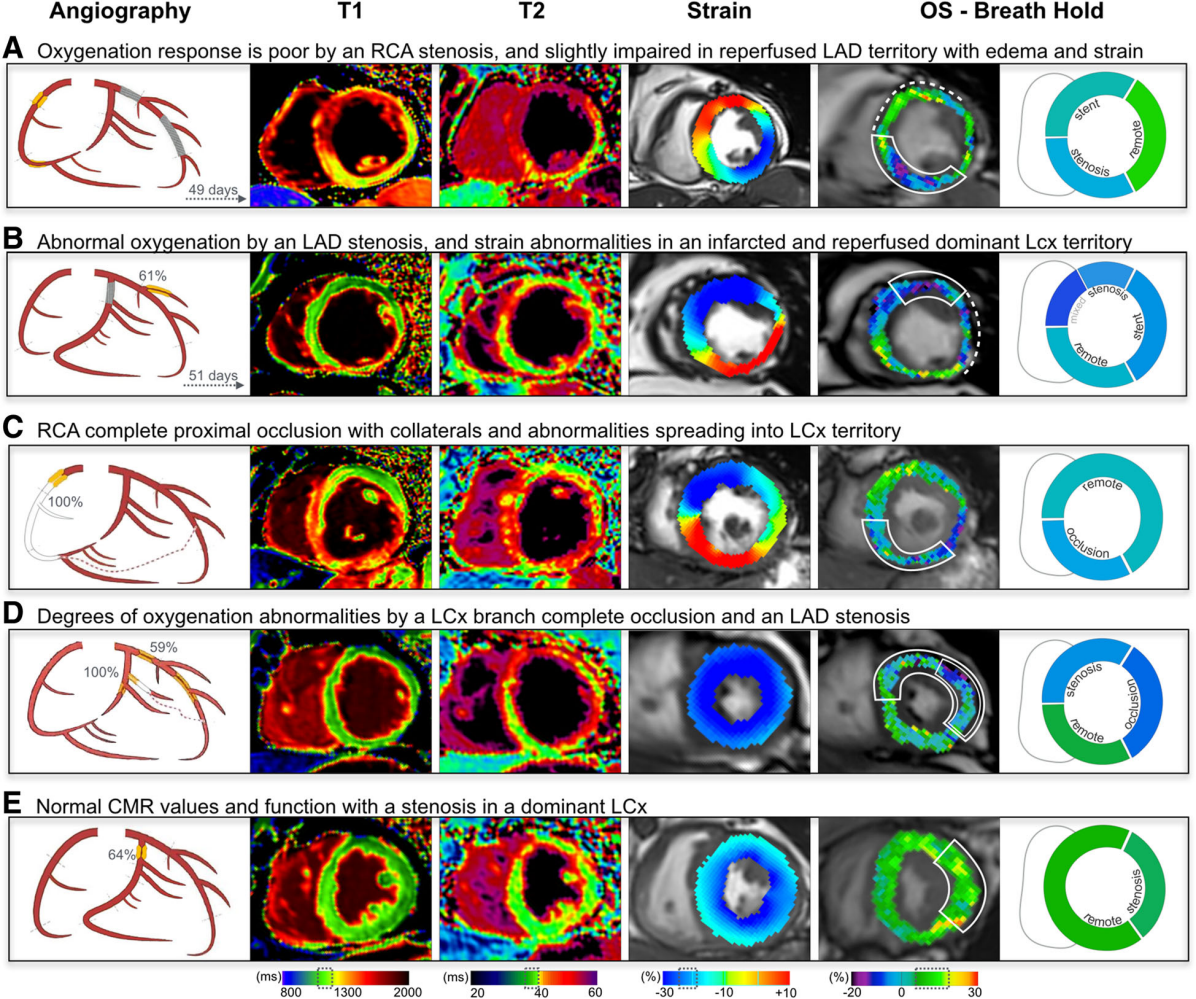
Different patterns of myocardial oxygenation response and ischemia/reperfusion injury. A pictogram of the angiography results is shown for the RCA, LCx and LAD and their major branches (left to right), and collateralizations (red dotted lines). For the CMR images, the normal range (mean + SD) of the healthy subjects is shown in the colour legends below, with strain and OS-CMR shown at end-systole, and T1 and T2 imaged in diastole. Patients **a** and **b** underwent primary PCI during the first visit and have reperfused vascular territories in addition to a stenosis. Patients **c**-**e** were scheduled for a later PCI or CABG, thus their index angiography was only diagnostic and there is no revascularized territory at the time of the CMR scan. Solid line boxes highlight the post-stenotic and dotted lines the reperfused territories. Detailed information for each case is provided in the Additional file 2


Breath-holding after hyperventilation consistently induced regional differences in both, stenosed (+ 0.5 ± 3.8%, *p* = 0.011) and reperfused territories (+ 0.7 ± 5.8%; *p* = 0.020), which showed a weaker response than remote territories (+ 3.8 ± 5.3%). Interestingly, in 5 (21%) of 24 patients with breath-holds of more than 30s, there was a global deoxygenation response during apnea, i.e. a SI decrease in all territories (exemplary patient shown in Fig. 2c). Despite global deoxygenation, as seen with the %-change colour overlay maps, a poorer oxygenation response in the post-stenotic territories could be visualized (Fig. 5 and the video in Additional file 1, with the explanation in Additional file 2).

**Additional file 1:** Video. (MP4 3019 kb)


### Relationship of CMR to demographics

In CAD patients a poorer oxygenation response to the breath-hold was associated with a younger age (*r* = 0.405, *p* = 0.049). From all the CMR data, no measurements were associated with the degree of stenosis defined by QCA. Similarly, most measurements were not associated with the days between the first PCI, except for the OS response to hyperventilation in reperfused segments (*r* = − 0.464, *p* = 0.026).

## Discussion

These results indicate that a combination of oxygenation-sensitive CMR with the combined breathing maneuver of hyperventilation and breath-holding may be a clinically feasible and safe diagnostic procedure to detect regional coronary vascular dysfunction associated with significant CAD. This was possible without the use of any pharmacological vasodilators or exogenous contrast agents. This is the first study using this diagnostic paradigm in a patient cohort with multi-vessel CAD. The combination of a preceding hyperventilation made this extended breath-hold feasible for 92% of the CAD patients to last at least 30 s, unmasking myocardium subtended to stenotic coronary arteries. No clinical symptoms indicative of myocardial ischemia were reported, and only minor transient general symptoms related to hyperventilation occurred.

### Global myocardial effects

In our healthy group, breathing maneuvers induced a homogenous oxygenation response throughout the myocardium, similar to previously published in healthy subjects, consisting of a drop in myocardial oxygenation with hyperventilation, and increased myocardial oxygenation during a breath-hold due to the associated vasodilation [14, 17]. In the CAD patients, this vaso-reactivity was globally blunted for both maneuvers.

### Regional myocardial oxygenation responses

The breath-hold induced a significant contrast in the myocardial oxygenation response between territories that were subtended to a stenosed coronary artery or a recently stented vessel. These showed a significantly poorer OS response than remote myocardium supplied by non-stenosed coronary arteries.

In this study, not only did the technique elicit clear differences in the OS-CMR breath-hold response between remote territory and myocardium affected by a stenosis, but there was also a consistent global abnormality in a portion of patients, where all territories revealed an oxygenation deficit (Fig. 2c). However in these patients, even despite global deoxygenation the decrease was more pronounced in myocardial segments with the associated coronary artery stenosis. In fact, patients with multi-vessel CAD can have balanced ischemia and may not show regional heterogeneity in diagnostic imaging stress tests. Usually a uniform response in some diagnostic tests results in difficulties creating an accurate diagnosis [18]. The global myocardial deoxygenation at OS-CMR unmasked these segments as diseased, which otherwise may have not been detected due to the uniformity of the myocardial response with other imaging modalities. However, this assumption requires validation in future studies by comparison to reference diagnostic tests. Outcome studies have linked impaired coronary flow reserve in both reperfused and reference vessels in multi-vessel disease as well, signifying global perfusion deficits and a higher cardiac mortality [19]. Our results call for studies looking not only at impaired flow reserve but also impaired oxygenation reserve and linking this impact of global abnormalities to clinical outcome.

As seen in Figs. 3 and 4, the remote territories showed variation with all the sequences. The variability in microvascular (dys-)function, collateralization and coronary steal, especially in multi-vessel CAD, can also produce variable results in remote myocardium [11, 20]. All these factors can act in a way that blood flow to the affected segments is either enhanced or diverted. Evidence for such an impact on perfusion results was found in a first-pass perfusion CMR study, in which perfusion deficits were not always completely consistent with coronary anatomy [21]. CMR myocardial mapping results have also indicated that remote myocardium may be subject to alterations after treatment of the culprit vessel, with high T1 in these territories of some patients, similar to what is observed in the current study [22]. Vascular dysfunction in myocardium subtended to patent coronary vessels may play a significant role in CAD patients.

While current analysis focuses on the vasodilatory component of the breath-hold, the assessment of hyperventilation alone may provide unique information about the vascular response. Hyperventilation was primarily used to induce vasoconstriction and allow a greater range in vasomotion to be assessed with the breath-hold, and to allow for a more feasible long breath-hold. When assessing the OS response to hyperventilation independently, patients had a globally attenuated response compared to healthy subjects, yet our small sample did not demonstrate regional significance in the determination of macrovascular disease. However, the response to this vasoconstrictive stimulus and the possible impact of microvascular dysfunction could provide a future direction in research.

### Feasibility of the protocol

**Fig. 6:**
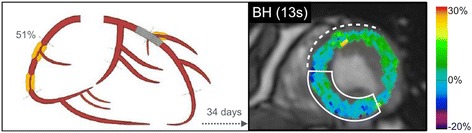
Myocardial oxygenation in truncated breath-holds. In this patient with a 51% RCA stenosis (solid line box) and reperfused LAD (dotted line marker), an extended breath-hold could not be performed. Yet, with the last image obtained only at 13 s, the RCA territory already shows an oxygen deficit in comparison to the increase of the remote territory

The clinical feasibility of a diagnostic procedure using hyperventilation and especially long breath-holds raise questions about the ability or willingness of patients to perform this, specifically maintain a breath-hold for 30s. Our data however show that in an elderly patient group with multi-vessel CAD, the mean breath-hold time was still 45 s following hyperventilation with only minor adverse effects reported. The advantage of this technique for the patient is that the patient remains in total self-control of the breathing maneuver, which can be aborted in the patient’s own discretion. We have previously shown that patients with obstructive sleep apnea could perform the breathing maneuvers with relatively similar breath-hold times [23]. Furthermore, a discernible difference between controls and patients was already detected at 15 s, which could also be visualized in the present study (see Fig. 2). Two patients could not hold their breath for an extended time (13 s and 19 s). However as shown in Fig. 6, myocardial oxygenation abnormalities could already be detected during this short breath-hold, matching the territory of the stenotic artery, yet it is undetermined if this early marker can be quantifiably reproducible.

### Myocardium post reperfusion

While the goal of the study was to assess myocardium subtended to a coronary artery stenosis, due to our recruitment method through staged PCI the majority of patients also had tissue that was reperfused on average 37 ± 11 days prior to the CMR. An interesting finding was that with the breath-hold in some patients, OS-CMR detected an attenuation in these reperfused territories, while in other patients there was a normal response. These heterogeneous findings were not unique to the OS-CMR, but were also observed in T1 mapping, T2 mapping and strain. The findings that some patients still had abnormal results despite reperfusion therapy may be explained by non-viable myocardium after previous infarction or ischemia-reperfusion injury. There was no significant correlation to the days between the CMR exam and revascularisation for the sequences except the OS-CMR during the hyperventilation stimulus. Other studies have addressed the success rate of PCI and an abnormal perfusion after revascularization is known [24]. Recent CMR studies have used T2 mapping in addition to T2* to show hemorrhage, microvascular obstruction and possibly scar exist after reperfusion [25]. This analysis was not one of the major end-points of our study, and we performed the exam within a sub-acute time-period of the intervention, and thus we cannot comment on long-term recovery of the reperfused myocardium.

### Comprehensive CMR exam

OS-CMR could be used as a part of a comprehensive CMR exam, including data on volumetry and function (cine images), as well as tissue characteristics (T1 and T2 maps). Along with strain analysis, native mapping sequences are now suggested as alternatives to contrast imaging for scar tissue [26, 27]. This could result in shorter protocols, and allow for more patients to undergo CMR exams. This includes patients who do not want to receive gadolinium contrast agents due to possible adverse effects, or those who are contraindicated for reasons such as renal failure, in which glomerular filtration rates of < 60 ml/min/1.73m^2^, which has been reported to be as high as 22% in a large cohort of stable CAD patients, with 6.9% experiencing severe chronic kidney disease (glomerular filtration rates of < 45 ml/min/1.73m^2^) [28].

### Limitations

In the present study, the severity of coronary artery stenosis was estimated by QCA measurements, rather than FFR, which is the current gold standard. Hemodynamic assessments such as FFR are not commonly performed at this site and were thus not available for the study. Another study using OS-CMR with adenosine as the vasodilator had shown a correlation to the degree of stenosis assessed by QCA, albeit weaker than with FFR [9]. Additionally, this study considered a stenosis with a diameter reduction of 50% as anatomically obstructive. While this threshold is commonly used as a cut-off for revascularization and has been linked with perfusion and oxygenation deficits [29], the FAME trial has shown that in multi-vessel CAD, visually assessed 50–70% stenosis did not routinely have functionally significant FFR readings [30]. Furthermore, as the study recruited patients during the staged PCI pathway, it was likely that the most severe lesion was treated in the primary visit, and that the less severe lesion remained stenosed for the subsequent CMR exam. Consequently, our results in post-stenotic territory could be under-appreciated due to a possible lack of a hemodynamically relevant stenosis and this could result in a larger variation of the OS response, yet this study does report a significant difference in comparison to remote myocardium. Our results are heavily based on the angiography classification and multiple factors can lead to discordance (see Fig. 5c), such as the presence of overlapping perfusion zones, collateralisation, as well as variation in patient coronary anatomy. Especially in the case of multi-vessel disease, the vasculature may adapt to the disease over time, and stray from the classical perfusion patterns.

It is important to note that in this study we did not perform contrast enhanced imaging to assess scar. In the reperfused territories we often observed a thin myocardium in the infarcted area with poor circumferential strain suggesting scar [27], yet there are no studies published that assess the effects of scar tissue on OS-CMR and breathing maneuvers specifically. Furthermore, in segments with a very thin wall (Fig. 5b), partial volume effects may limit the accuracy. OS images are also susceptible to artifacts if present within regions of interest, which can lead to lesser image quality and segmental exclusion (Fig. 2b). Consequently, we also did not image the apical slice as it is the most prone to artifacts and partial volume effects [9], and has a large variation in vascular territory dependence. Additionally, the acquisition time of the current sequence is not short enough to yet obtain full-coverage of the heart and reliably catch the highly responsive first 15 s of the breath-hold.

During the study design, we chose to recruit younger participants in good general health since they were most likely to have minimal microvascular or other cardiovascular dysfunction, The oxygenation response of these young healthy subjects was similar to our previous publication, which recruited an older healthy population [23]. We recognize that differences in CMR results between the CAD group and the healthy subjects could also be caused or influenced by numerous other factors such as age, body mass index, and use of medications and this is a limitation of the study. However, these are not factors that can be accurately assessed in our small sample of healthy participants. Yet, significant regional differences were still observed within the CAD myocardium itself, demonstrating that the coronary stenosis is a major contributing factor. This study is limited by the small sample size, and results of such studies are prone to larger margins of error. It was not powered to assess the diagnostic value of this test to diagnose the severity of coronary artery disease. Larger, preferably multi-center trials are needed to assess the actual clinical utility of OS-CMR in clinical settings, including the feasibility of the technique in a larger population, and relate these findings to clinical outcome.

## Conclusion

Our results indicate that a combined maneuver of hyperventilation and subsequent breath-holding is a feasible method to elicit a coronary vasomotor response in CAD patients associated with minimal side effects. In conjunction with OS-CMR, the breathing maneuvers can unmask oxygenation deficits in myocardium subtended to stenotic coronary arteries.

## Additional files

Additional file 2: Explanation of the video file. (PDF 102 kb)

## Abbreviations

bSSFP: balanced steady state free precession
CABG: Coronary artery bypass graft
CAD: Ccoronary artery disease
CI: Cardiac index
CMR: Cardiovascular magnetic resonance
dHb: Deoxyhemoglobin
DS: Diameter stenosis
EDV_I_: End-diastolic volume index
EF: Ejection fraction
ESV_I_: End-systolic volume index
FFR: Fractional flow reserve
Hb: Hemoglobin
ICC: Intraclass correlation coefficient
LAD: Left anterior descending
LCx: Left circumflex
LV: Left ventricular/left ventricle
Mass_I_: Mass index
NSTEMI: Non-ST-elevation myocardial infarction
OS: Oxygenation-sensitive
PCI: Percutaneous coronary intervention
QCA: Quantitative coronary angiography
RCA: Right coronary artery
SD: Standard deviation
SI: Signal intensity
STEMI: ST-elevation myocardial infarction
