# Supplementary material for: Feasibility of cardiovascular magnetic resonance to detect oxygenation deficits in patients with multi-vessel coronary artery disease triggered by breathing maneuvers
Source: J Cardiovasc Magn Reson. 2018 May 7;20:31. doi: 10.1186/s12968-018-0446-y (PMC5937049; doi:10.1186/s12968-018-0446-y)
Supplement: Supplementary file 2 — Explanation of the video file. (PDF 102 kb) [file 12968_2018_446_MOESM2_ESM.pdf]

Feasibility of cardiovascular magnetic resonance to detect oxygenation deficits in patients with multi-vessel coronary artery disease triggered by breathing manoeuvres.

Kady Fischer PhD.[1,2,3]; Kyohei Yamaji, MD[4]. Silvia Luescher[1], Yasushi Ueki, MD[4]. Bernd Jung, PhD[3]. Hendrik von Tengg-Kobligk, MD[3], Stephan Windecker, MD[4]. Matthias G. Friedrich, M.D[2,5], Balthasar Eberle. MD[1], Dominik P. Guensch, MD[1,3*]

[1] Department Anaesthesiology and Pain Therapy, Inselspital, Bern University Hospital, University of Bern, Bern, Switzerland
[2] Research Institute of the McGill University Health Centre, Montreal, Canada
[3] Department of Interventional and Paediatric Radiology, Inselspital, Bern University Hospital, University of Bern, Switzerland
[4] Department of Cardiology, Inselspital, Bern University Hospital, University of Bern, Switzerland
[5] Department of Cardiology, Heidelberg University Hospital, Heidelberg, Germany

Clinical Trials Identifier: NCT02233634

***Address for correspondence:** Dominik P. Guensch, MD, DESA, Department of Anaesthesiology and Pain Therapy, Bern University Hospital, Inselspital, University of Bern, 3010 Bern, Switzerland. Tel.: +41 (0)316323965. Fax: +41 (0)31-632-0554. E-mail: dominik.guensch@gmail.com

Table of Contents

Figure 5: Detailed case information.. 2

Video: Myocardial oxygenation over the breath-hold 4

Figure 5: Detailed case information

A. In a 74 year-old female with an initial STEMI, the territory currently affected by an RCA stenosis shows that during the vasodilatory breath-hold there is an oxygenation deficit in the territory subtended to the stenosed vessel. In this territory there is a high T2 value, while T1 and strain are moderately abnormal, with poorer measurements bordering the LAD territory. OS is normal in the reperfused myocardium, there is poor strain and T1 and T2 show very high signal in the LAD territory still 49 days after the PCI.

B. For this 64 year old male patient referred for a STEMI, there is a clear infarction in the LCx territory. The oxygenation response is poor in both the territory distal to the stenosed and reperfused vessel. However, measurements in the reperfused territory are regarded with caution as wall thickness measures only 3mm at end-systole, and voxels can be prone to partial volume effects.

C. Angiography for a 63 year-old female revealed a complex coronary system, as although the RCA is fully occluded proximally in the vessel, it was determined that collaterals partially compensate for the vessel. As seen with CMR, there is a significant strain abnormality and high T1 centred in the RCA territory, but spreading into the septum and neighbouring LCx territory. High T2 and an OS deficit during the breath-hold also spread beyond the RCA, however in the centre of the RCA territory at the inferior insertion point, there is no T2 enhancement, and the OS response in this region of interest was 0.4%, possibly signifying this area could be scar with no viable myocardium. In all images the anterior wall is fairly normal. This patient demonstrates how angiographic

results with the assigned affected segments may be incongruent with the actual observed contractility and oxygenation impairment.

D. A 62 year-old male had multi-vessel disease with collaterals. The oxygenation results during the breath-hold in this patient demonstrate different degrees of myocardial oxygenation responses. In the lateral wall subtended to a fully occluded LCx branch, there is a large decrease in OS, while in the neighbouring territory subtended to partially occluded vessels, the oxygenation response is attenuated, but the response is not as poor as in the territories subtended to the full occlusion. Furthermore, there is high T2 signal in the partially occluded areas, but not in the tissue subtended to the occlusion. This suggests that there may be some degree of possible repetitive ischemia/reperfusion-like events. While at rest perfusion may be sufficient for normal tissue oxygenation, however with physical activity there may be transient ischemic periods, with reperfusion injury occurring after the patient goes back to a resting period. Strain and T1 were normal.

E. A 68 year-old male was scheduled for CABG surgery, primarily because of ST-depression during a physical stress test and he has insulin-dependent diabetes. Despite a proximal LCx stenosis, CMR measurements were within normal range, and EF was 74% with a cardiac index of 2.3L/min/m^2.

Video: Myocardial oxygenation over the breath-hold

A time-lapse of the myocardial oxygenation response over the duration of the breath-hold is shown for the cases shown in Figure 2. The myocardial oxygenation response is shown using %-change pixelated maps masked onto an original OS-cine for each measurement obtained in the breath-hold, commencing with a blue contour representing 0%.

Healthy Control: The myocardial response of a healthy 24 year-old male shows a homogeneous increase in signal for the breath-hold, represented by green and yellow pixels. The average response for healthy controls is shown on the graph (green shaded curve), in which the myocardial response increases rapidly at the beginning of the breath-hold and is then maintained throughout apnea.

CAD Patient with a Regional Response: In the middle is a 73 year-old male patient with an LAD stenosis (70%DS), and a revascularized RCA (29 days post-PCI with a stent) who demonstrated a regional response. In the post-stenotic (blue dotted line on graph) and reperfused territories, oxygenation decreased by the early time-point at 12s and continued to decrease further throughout the breath-hold, represented by blue and purple pixels. On the other hand, the remote territory from a patent LCx responded with an oxygenation increase, as shown on the graph by the green dotted line. A portion of the inferolateral wall was removed due to image artefact.

CAD Patient with a Globally Abnormal Response: The 51 year-old male patient on the right demonstrated a global dysfunction in which all territories had an oxygenation deficit with a signal decrease throughout the breath-hold (purple dotted line on graph representing the response of the entire myocardium). This patient had a distal RCA stenosis (51%DS), with a stent on the LAD and the first diagonal branch of the LAD (49 days post-PCI).
